# Risk Factors for *Salmonella*, Shiga Toxin-Producing *Escherichia coli* and *Campylobacter* Occurrence in Primary Production of Leafy Greens and Strawberries

**DOI:** 10.3390/ijerph120809809

**Published:** 2015-08-18

**Authors:** Siele Ceuppens, Gro S. Johannessen, Ana Allende, Eduardo César Tondo, Fouad El-Tahan, Imca Sampers, Liesbeth Jacxsens, Mieke Uyttendaele

**Affiliations:** 1 Laboratory of Food Microbiology and Food Preservation (LFMFP), Department of Food Safety and Food Quality, Faculty of Bioscience Engineering, Ghent University, Ghent 9000, Belgium; E-Mails: Siele.Ceuppens@UGent.be (S.C.); liesbeth.jacxsens@ugent.be (L.J.); 2 Norwegian Veterinary Institute, P.O. Box 750 Sentrum, 0106 Oslo, Norway; E-Mail: gro.johannessen@vetinst.no; 3 Research Group on Quality, Safety and Bioactivity of Plant Foods, Department of Food Science and Technology, CEBAS-CSIC, 30100 Murcia, Spain; E-Mail: aallende@cebas.csic.es; 4 Laboratório de Microbiologia e Controle de Alimentos, Instituto de Ciência e Tecnologia de Alimentos, Universidade Federal do Rio Grande do Sul (ICTA/UFRGS), Av. Bento Gonçalves, 9500, Prédio 43212, Campus do Vale, Agronomia, Cep. 91501-970 Porto Alegre/RS, Brazil; E-Mail: tondo@ufrgs.br; 5 Royal International Inspection Laboratories (RIIL), Suez 43111, Egypt; E-Mail: fouadeltahan@yahoo.co.uk; 6 Laboratory of Food Microbiology & Biotechnology, Department of Industrial Biological Sciences, Faculty of Bioscience Engineering, Ghent University, Kortrijk 8500, Belgium; E-Mail: Imca.Sampers@UGent.be

**Keywords:** primary production, *E.coli*, index, climate, logistic regression, risk factors

## Abstract

The microbiological sanitary quality and safety of leafy greens and strawberries were assessed in the primary production in Belgium, Brazil, Egypt, Norway and Spain by enumeration of *Escherichia coli* and detection of *Salmonella,* Shiga toxin-producing *E. coli* (STEC) and *Campylobacter*. Water samples were more prone to containing pathogens (54 positives out of 950 analyses) than soil (16/1186) and produce on the field (18/977 for leafy greens and 5/402 for strawberries). The prevalence of pathogens also varied markedly according to the sampling region. Flooding of fields increased the risk considerably, with odds ratio (OR) 10.9 for *Salmonella* and 7.0 for STEC. A significant association between elevated numbers of generic *E. coli* and detection of pathogens (OR of 2.3 for STEC and 2.7 for *Salmonella*) was established. Generic *E. coli* was found to be a suitable index organism for *Salmonella* and STEC, but to a lesser extent for *Campylobacter*. Guidelines on frequency of sampling and threshold values for *E. coli* in irrigation water may differ from region to region.

## 1. Introduction

Fresh produce is part of a healthy diet and its consumption should be further encouraged. Daily consumption of five or more portions of fruits or vegetables decreases the risk of heart disease and stroke [1,2] and consumption of whole fruits lowers the risk of diabetes [3]. However, most fruits and many vegetables such as leafy greens are typically consumed raw. If these are microbiologically contaminated they also present an increased risk for foodborne illness. Several outbreaks illustrate that the microbial safety of fresh produce should not be neglected. *E. coli* O157:H7 outbreaks occurred in the US with strawberries in 2011 [4], romaine lettuce in 2011 [5], bagged spinach in 2006 [6], as well as an outbreak of *Salmonella* with peppers in 2008 [7]. In Europe a number of cases of *E. coli* 0157 were epidemiologically linked to fresh produce including watercress in England [8], iceberg lettuce in Sweden [9] and lettuce in Iceland and the Netherlands [10]. Another notorious incident was the *E. coli* O104 outbreak with sprouted fenugreek seeds in 2011 in Germany and the rest of Europe [11]. Leafy greens eaten raw as salads were involved in seven salmonellosis outbreaks reported in the EU in the period 2007–2011, involving 268 human cases in total [12]. *Campylobacter* is the most important cause of bacterial gastroenteritis reported cases in EU and is usually associated with broiler meat [13]. However, apart from *Salmonella* and Shiga toxin-producing *E. coli* (STEC)*, Campylobacter* has been highlighted as a relevant microbial risk for raw vegetables, fruits and minimally processed packaged salads [14,15]. *Campylobacter* is a known water borne pathogen [16,17] and often present in wild birds, thus with potential of fecal contamination to crops growing in the fields, as was reported in an outbreak of campylobacteriosis associated with peas [18]. Domestic and wild animals are reservoirs of *E. coli* O157 and *Salmonella* in the agricultural production environment and may contaminate fresh produce on the field, either directly or via contaminated agricultural water, as illustrated by several recent outbreaks [4,7,8,9].

Washing, including washing in water with sanitizers, will not accomplish more than 2 log reduction of bacteria (including pathogens) present on fresh produce [19,20,21,22,23,24,25,26]. In addition, the washing procedure may damage sensitive products, such as berries, thereby decreasing the quality and shelf life by increasing the sensitivity to spoilage and mold growth [27,28]. Profound knowledge of the contamination sources and pathways for introduction of bacterial pathogens in primary production of fresh produce is needed to focus on prevention of contamination events [29]. Irrigation water quality is of major importance for fresh produce quality, since it may be both a source and route of microbial contamination [30,31,32,33]. In case manure is used as an organic fertilizer, control of the composting process is also a critical point [32]. Combination of cattle rearing and fresh produce production is identified as a potential risk factor [34,35]. Climatic factors, *i.e.* increased temperatures and flooding events, were shown to be associated with a decreased microbiological quality and safety of leafy greens [30,32,36]. Most of these studies focused on one particular geographical region. The main objective of the present study is to investigate whether and which factors could be identified as universal risk factors for pathogen contamination of fresh produce across farms in various countries with variable climate and agro-technical management practices. For this purpose leafy greens, strawberries and their primary production environment (soil, water, contact surfaces) were analyzed for the presence of *Salmonella*, STEC, *Campylobacter* and the amount of generic *E. coli* using a similar sampling plan at a variety of farms in Belgium, Brazil, Egypt, Norway and Spain within the framework of the European Veg-i-Trade project, executing research on the topic of microbiological (and chemical) safety of fresh produce in a global context.

## 2. Experimental Section

### 2.1. Sampling Plan

In total, 3330 samples were taken from contact surfaces (524) including boxes, hands, blades, conveyers belts and tables, fertilizer (72), leafy greens (824) including lettuce, spinach and basil, strawberries (170), seeds (54), soil (1037) and water (649) including irrigation water from the source or reservoir, the tap, sprinkler or dripper and rinsing water for harvested crops on 45 farms in five countries (Belgium, Brazil, Egypt, Norway and Spain) [30,31,32,34,36,37,38,39] (Table 1). In the case of farms producing leafy greens, the sampling was repeated throughout the crop growth cycle: at planting, two weeks before harvest, one week before harvest and at harvest. In case of strawberries, the multiple sampling rounds were conducted over the production season, of which the timing depended on the country. Contact surfaces were swabbed: an area of 50 cm² or the whole hand surface, 200 g of fertilizer was taken 200 to 300 g soil samples were taken (usually three were pooled but not in all studies), three crops of lettuce were pooled, 1 kg of strawberries was sampled and three samples of 100 g spinach were pooled and 5 L irrigation or rinse water was taken. After mixing, subsamples of 25 g for solid samples and 25 to 1000 mL in case of water (volume depending on the microbial load) were used for pathogen detection.

**Table 1:** Overview of the samples taken per country and per fresh produce type.

Country	Product	Farms	Visits	Reference	Sample Types	Sampling Time				Total Samples
						Planting	Two Weeks before Harvest	One Week before Harvest	Harvest	
Belgium	Lettuce	8	3	Holvoet *et al.* (2014) [30]	Contact surfaces	0	0	0	104	104
					Lettuce	23	69	69	126	287
					Soil	126	69	69	69	333
					Water	0	37	36	47	120
					Total	149	175	174	346	844
Belgium	Strawberry	6	4	Delbeke *et al.* (2015) [34]	Contact surfaces					57
					Soil					48
					Strawberry					72
					Water					78
					Total					255
Brazil	Lettuce	6	1	Ceuppens *et al.* (2014) [32]	Contact surfaces	0	0	0	36	36
					Fertilizer	18	0	0	0	18
					Lettuce	6	18	18	33	75
					Soil	24	18	18	18	78
					Water	12	12	12	17	53
					Total	60	48	48	104	260
Egypt	Lettuce	6	1	Abdel-Moneim *et al.* (2014) [31]	Lettuce				18	18
					Soil				6	6
					Water				6	6
					Total				30	30
Egypt	Strawberry	6	1	Abdel-Moneim *et al.* (2014) [31]	Soil					6
					Strawberry					18
					Water					6
					Total					30
Norway	Lettuce	6	3	Johannessen (2015) [39]	Contact surfaces	0	0	0	31	31
					Lettuce	54	45	54	54	207
					Soil	63	45	54	54	216
					Water	0	14	20	18	52
					Total	117	104	128	157	506
Norway	Strawberry	4	4	Johannessen *et al.* (2015) [38]	Contact surfaces					80
					Soil					80
					Strawberry					80
					Water					16
					Total					256
Spain	Lettuce	2	3	Castro-Ibanez *et al.* (2015) [36]	Lettuce					21
					Soil					30
					Water					18
					Total					69
Spain	Spinach	3	3	Castro-Ibanez *et al.* (2015) [37]	Contact surfaces	0	0	0	216	216
					Fertilizer	54	0	0	0	54
					Spinach	0	54	54	108	216
					Seeds	54	0	0	0	54
					Soil	78	54	54	54	240
					Water	0	102	102	96	300
					Total	186	210	210	474	1080
**Overall**	**−**	**45**	**−**	**This study**	**Contact surfaces**					**524**
					**Fertilizer**					**72**
					**Leafy greens**					**824**
					**Seeds**					**54**
					**Soil**					**1037**
					**Strawberry**					**170**
					**Water**					**649**
					**Total**					**3330**

### 2.2. Microbiological Analyses

Details of the methods used for sampling and microbial analysis in the various countries can be found in prior description of these studies on a country level *i.e.* Belgium [30,34], Brazil [32], Egypt [31], Norway [38] and Spain [36,37]. Generic *E. coli* was enumerated in all studies and in all of the 3330 samples by equivalent methods including ISO 9308-1:2000 [40], APHA 1998 [41], US-EPA Standard Method 9222 D/G [42], Colilert**^®^**-10 Test kit, Quanti-Tray™/2000 and Chromocult**^®^** Coliform Agar in water samples and by RAPID’*E. coli* 2/Agar, 3M™ Petrifilm™ Select *E. coli*, 3M™ Petrifilm™ *E. coli*/Coliform, Chromocult**^®^** Coliform Agar and NMKL 125:2005 [43] for other sample types. Not all samples were also analyzed all the time for all pathogens as the capacity to analyze pathogens differed among the countries involved. In total 1605 samples (48.2%) were analyzed for *Salmonella* by equivalent methods including ISO 19250:2010 [44], ISO 6579:2002 [45] or NMKL 71:1999 [46] either as standard procedure or only as a subsequent method for isolation of the pathogen after prior screening for *Salmonella* using the GeneDisc**^®^** PCR test kit. In total*,* 509 samples were analyzed for *Campylobacter* (15.3%) by either ISO 17995:2005 [47], ISO 10272-1:2006 [48] or NMKL 119:2007 [49]. In total, 1545 samples were analyzed for Shiga toxin producing *E. coli* (STEC) (46.4%) either by ISO 16654:2001 [50] for STEC O157 or more broadly for non-O157 STEC using GeneDisc**^®^** PCR screening for the simultaneous occurrence of *stx1/2* toxin genes and *eae/aggR* adhesion genes, followed by isolation from presumptive STEC positive samples by plating on ChromID and CT-SMAC using the approach described in ISO 13136:2012 [51]. Positive PCR results were followed by culture isolation of the STEC strain. The presence of the virulence genes in the isolate were confirmed by PCR.

### 2.3. Agro-Technological Practices and Information on Climatic Conditions

Agro-technological practices were assessed during the farm visit by visual inspection and a questionnaire interview (e.g., as described by [32] and [35]). Climatic parameters were retrieved from the closest weather station. Flooding was defined as an event of excessive rainfall causing the fields to be inundated with accumulated rain water and/or water from overflowing natural water bodies such as nearby rivers within one week of sampling.

### 2.4. Statistical Analyses

All analyses were performed with SPSS Statistics version 21 at a significance level of 5 % (*p* = 0.050). The 95% confidence intervals for pathogen prevalence were calculated according to the Wilson score method without continuity correction [52]. Significant differences in the prevalence of pathogens were determined with the Mann-Whitney U test for continuous variables (*E. coli* counts and climatic parameters) and with the Chi-squared test of independence for categorical variables (agro-technical parameters). The presence/absenceof pathogens determined by culture was also modelled by multiple logistic regression according to the purposeful selection method [53]. Briefly, the significant main effects were determined by adding all covariates univariably in the logistic regression. All those with *p* < 0.250 were included as potential main effects in one multivariable model on which stepwise backward likelihood ratio selection was performed. All omitted variables were added one-by-one to the obtained model and those with *p* < 0.050 were kept. The assumption of linearity was checked for all continuous variables by adding the quadratic term as a main effect to the regression model. Then, all possible interactions were tested univariably and those with *p* < 0.250 were added together for forward LR model selection. Main effects were never eliminated, even if they lost their significance in the presence of the interaction. The Hosmer and Lemeshow test was used to check if the model fitted well to the data. The Cook’s distance and standardized residuals were plotted to check for highly influential data points and biases in the predictions. Sensitivity and specificity of the model were checked by Receiver Operating Characteristic (ROC) curve analysis. ROC curves are graphical representations of the sensitivity and specificity for each possible cut-off value of the test variable [54]. The area under the ROC curve (AUC) is the summary statistic which gives an idea of the overall diagnostic performance of the test, with the AUC ranging from 0.5 for meaningless to 1.0 for perfection. In our case, the AUC indicates the ability to predict the presence of pathogens.

## 3. Results and Discussion

### 3.1. Occurrence of Pathogens and Generic E. coli

Within the framework of the EU FP7 Veg-i-Trade project the microbiological sanitary quality and safety of leafy greens and strawberries were assessed in the primary production in Belgium, Brazil, Egypt, Norway and Spain by the enumeration of *E. coli* and the detection of *Salmonella*, STEC and *Campylobacter* in these products and in their primary production environment. Although a substantial number of analyses were carried out, only few bacterial pathogen detections were observed within the combined data set.

The overall prevalence of *Salmonella* in all samples analyzed (n = 1605) was 2.5% (95% confidence interval (CI): 1.8%–3.4%) (Table 2). *Salmonella* occurred most frequently in fertilizers (7.4% (2/27)), probably due to insufficient control of the composting process of manure used as organic fertilizer [55]. Irrigation water was second most contaminated (3.1% (12/387)) with *Salmonella*, probably because monitoring of the microbial water quality, and if necessary application of water treatment, was not (widely) applied by farmers [35]. The prevalence in the other sample types was similar, between 1.8% and 2.9%. This relatively high prevalence in fresh produce was caused by the study in Egypt, sampling small scale farmers providing local market, which showed a considerably higher incidence of *Salmonella* in fresh produce than the other studies. All (5/5) of the *Salmonella* positive strawberries and seven out of the 12 *Salmonella* positive lettuce samples were from Egypt [31]. STEC was isolated by culture in 0.7% of all samples (n = 1545) (95% CI: 0.4%–1.3%), most often from irrigation water samples. It should be noted that positive PCR signals for both *stx* and *eae* genes were obtained for much more samples (68 positives), but subsequent culture confirmation of STEC proved difficult (11 isolates obtained) [34,38]. It has been acknowledged that the culture isolation procedures for STEC are difficult and prone to failure, in particular in samples with high numbers of competing microbiota [56,57,58]. Moreover, STEC strains may easily loose *stx* genes, as early as during the first sub-cultivation step [59]. In this manuscript, only culture confirmed results were regarded as positive. *Campylobacter* was isolated at an overall prevalence of 8.6% (95 CI: 6.5%–11.4%) (n = 509), again mostly from water sources. Pathogens were mainly isolated from the production environment rather than from the leafy greens or strawberries themselves sampled at these fields, as noted by other studies [14,60,61]. No pathogens were detected on seeds (n = 27) and contact surfaces (n = 72) such as hands, boxes used at harvest, *etc.*

The detection of pathogens varied according to the geographical region. Amongst other reasons such as differences in environmental pressure and climate, this may be affected by the different status of implementation of good agricultural practices and national measures, guidelines or support available to these farmers involved [62,63].

**Table 2:** Pathogen prevalence per sample type.

*Salmonella*	Analyses	Positives	Prevalence (%)	95 % Confidence Interval	
Contact surfaces	36	0	0.0	0.0	9.6
Fertilizer	27	2	7.4	2.1	23.4
Seeds	9	0	0.0	0.0	29.9
Strawberry	170	5	2.9	1.3	6.7
Leafy greens	377	10	2.7	1.4	4.8
Soil	599	11	1.8	1.0	3.3
Water	387	12	3.1	1.8	5.3
Total	1605	40	2.5	1.8	3.4
**Shiga toxin-producing *E. coli* (STEC)**	Analyses	Positives *	Prevalence (%) *	95 % confidence interval *	
Contact surfaces	36	0 (0)	0.0	0.0	9.6
Fertilizer	27	0 (0)	0.0	0.0	12.5
Seeds	9	0 (0)	0.0	0.0	29.9
Strawberry	152	0 (0)	0.0	0.0	2.5
Leafy greens	359	0 (1)	0.0	0.0	1.1
Soil	587	5 (34)	0.9 (5.8)	0.4 (4.2)	2.0 (8.0)
Water	375	6 (33)	1.6 (8.8)	0.7 (6.3)	3.4 (12.1)
Total	1545	11 (68)	0.7 (4.4)	0.4 (3.5)	1.3 (5.5)
***Campylobacter***	Analyses	Positives	Prevalence (%)	95 % confidence interval	
Strawberry	80	0	0.0	0.0	4.6
Leafy greens	241	8	3.3	1.7	6.4
Water	188	36	19.1	14.2	25.4
Total	509	44	8.6	6.5	11.4
**All pathogens**	Analyses	Positives	Prevalence (%)	95 % confidence interval	
Contact surfaces	72	0	0.0	0.0	5.1
Fertilizer	54	2	3.7	1.0	12.5
Seeds	18	0	0.0	0.0	17.6
Strawberry	402	5	1.2	0.5	2.9
Leafy greens	977	18	1.8	1.2	2.9
Soil	1186	16	1.3	0.8	2.2
Water	950	54	5.7	4.4	7.3
Total	3659	95	2.6	2.1	3.2

* Positive results were culture confirmed, between brackets the PCR positive results are given.

In general (*i.e.*, taken all samples together), isolation of *Salmonella*, STEC and *Campylobacter* occurred from samples which also contained significantly higher counts of generic *E. coli* (*p* < 0.001, *p* = 0.046 and *p* < 0.001, respectively). When considering the results separately per sample type, *E. coli* also performed well as an index organism because the presence of pathogens was usually significantly associated with elevated *E. coli* numbers*,* except for fertilizer samples in association with *Salmonella* and soil samples with STEC (Table 3). The performance of *E. coli* as an index organism was better (AUC > 0.8) for *Salmonella* than for STEC and *Campylobacter*, in all sample types. Moreover, *E. coli* had a better functionality to serve as an index organism in water samples than in soil and fresh produce (leafy greens or strawberries) samples in the present study. Remarkably, although the isolation of STEC was significantly more frequent from water samples with elevated generic *E. coli* levels, this was not the case in soil, where generic *E. coli* had no significant predictive ability for STEC. The relation of generic *E. coli* with a pathogen may thus also vary on the environmental setting (*i.e.*, the sample type). The presence of *Campylobacter* in fresh produce exhibited a significant but reverse association with *E. coli*: this pathogen was isolated more frequently when no or low levels of *E. coli* were present. In general, it should be noted that even when significant and positive correlations existed, these were never completely consistent. Detection of 100 % of the pathogen positive samples was not possible with any *E. coli* threshold value, because pathogens were occasionally isolated from samples which were negative for generic *E. coli*. To illustrate: in our study, 15% (6/40) of all samples positive for *Salmonella* had *E. coli* numbers below the detection limit (<10/g, except for the Spanish analyses and <1/100 mL for all water analyses) and this was 23 % (10/44) for *Campylobacter* (<10/g or <1/100 mL for all analyses). For STEC, no samples were positive by culture (0/11) when generic *E. coli* was below the detection limit. Since the detection limit for solid samples was tenfold higher in the Spanish study [37], for three *Salmonella* positive samples *E. coli* was < 100/g instead of 10/g.

**Table 3:** Receiver Operating Characteristic (ROC) curve analysis of each index for each pathogen per sample type, showing the area under the curve (AUC) and number of samples (N) on which the ROC analysis was performed.

Predictor	*Salmonella*	STEC	*Campylobacter*
**Overall**			
Logistic regression	AUC = 0.927 (n = 1530)	AUC = 0.870 (n = 1545)	AUC = 0.878 (n = 476)
*E. coli*	AUC = 0.838 (n = 1605)	AUC = 0.665 (n = 1545)	AUC = 0.697 (n = 509)
**Produce**			
*E. coli*	AUC = 0.910 (n = 547)	No positives (n = 511)	AUC = 0.135 (n = 321)
**Water**			
*E. coli*	AUC = 0.820 (n = 387)	AUC = 0.850 (n = 375)	AUC = 0.763 (n = 188)
**Soil**			
*E. coli*	AUC = 0.847 (n = 599)	Not significant (n = 587)	No data (n = 0)

**Figure 1:**
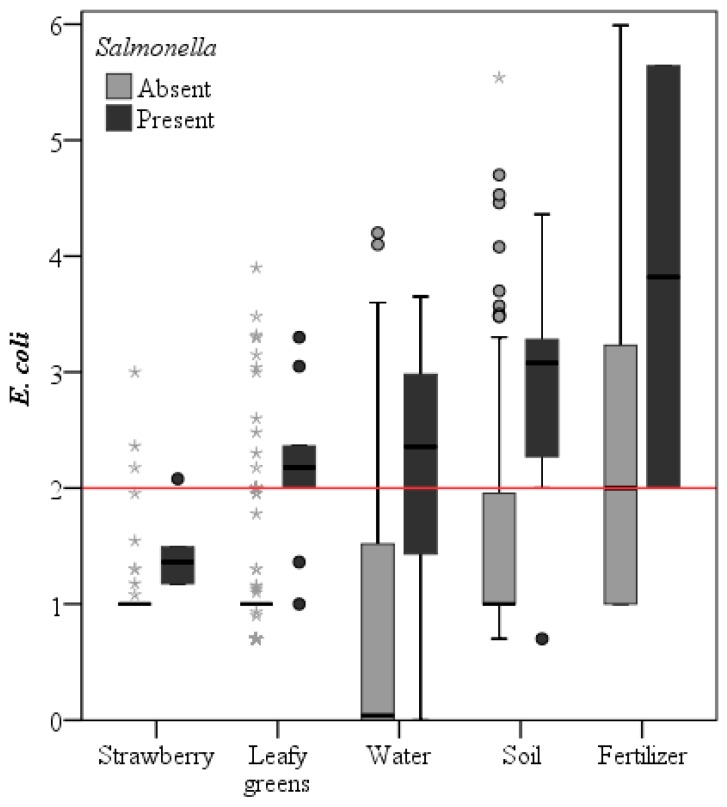
Pathogens were associated with higher generic *E. coli* counts (in log CFU/g or 100 mL), exemplified here by showing all *Salmonella* analyses per sample type (except for seeds and contact surfaces, since these were always negative). The horizontal red line indicates the threshold of 100 CFU *E. coli* per gram or 100 mL to show the potential impact of setting this value as a limit. Outliers are presented as circles (1.5 to 3 times the interquartile range below the 25th percentile or above the 75th percentile) or as asterisks (more than three times the interquartile range).

When data processing is done according to the investigated regions and the sample type, interesting findings can be reported (Figure 1). If the threshold value is put at 100 *E. coli* per g leafy greens or strawberries, between 50% (Egypt and Spain) and 100% (Brazil) of the fresh produce samples which tested positive for *Salmonella* would be identified by exceeding this *E. coli* threshold. But at the same time this limit would affect in total 0.6% (Belgium) to 25% (Egypt) of the fresh produce samples, most of which would be false-positive, resulting in food waste and an economic burden of loss or further testing for pathogens. Given the low counts of generic *E. coli* on strawberries, the threshold of 100 CFU/g would be too high; 15 CFU/g would be more appropriate. If the threshold value is put at 100 *E. coli* per 100 mL irrigation water, between 0% (Belgium) and 100% (Egypt and Norway) of water containing *Salmonella* would be rejected for irrigation, but this limit would result in a high rejection rate of the currently used water sources, ranging from 19% (Belgium) to 83% (Egypt). Pathogens present in irrigation water may not be transferred to the fresh produce if the contact between water and produce is restricted, for example by drip irrigation, and the threshold value for acceptable water quality may be set higher if such risk reducing strategies are employed [64]. Alternatively, to improve the microbiological quality of the water, the water could be subjected to various treatments (filtration, chemical decontamination, UV irradiation, sonication, *etc.*) before application as irrigation water [65,66].

### 3.2. Risk Factors for Increased Likelihood of Finding Pathogens

A number of agro-technical factors were investigated individually for a significant relation with the occurrence of pathogens (Table 4). Specific countries, elevated generic *E. coli* numbers, flooding events and specific irrigation water sources (categorized as surface water, collected rainfall water, borehole water or municipal potable water) were associated with a higher probability of occurrence of all pathogens: *Salmonella,* STEC and *Campylobacter*. *Salmonella* was most often found (6.2%, 32 positive out of 513 samples) when surface water was the irrigation water source, while *Campylobacter* (20.8%, 30/144) and STEC (1.7%, 10/581) were more often isolated when collected rainfall water was the irrigation water source. Specific sample types and elevated average daily temperatures at the day of sampling were significantly linked with the presence of *Salmonella* and *Campylobacter* but not with STEC. Increased likelihood of STEC and *Campylobacter* was observed in case farmers combined cultivation of fresh produce crops with animal production. The use of (insufficiently) composted manure as a fertilizer and the use of flood irrigation was associated with increased *Salmonella* prevalence. Lower precipitation at the day of sampling, absence of any disinfection treatment of the irrigation water and storage of irrigation water in open reservoirs (ponds) was correlated with elevated *Campylobacter* isolation rates.

**Table 4:** List of factors which were univariably investigated in logistic regression for significance (see *p*-value, significance at the 5% level is indicated by grey boxes).

Factors	*Salmonella*	STEC	*Campylobacter*
Country (Belgium, Brazil, Egypt, Norway, Spain)	*p* < 0.001	*p* = 0.004	*p* < 0.001
Generic *E. coli* (log CFU/g or 100 mL)	*p* < 0.001	*p* < 0.001	*p* < 0.001
Irrigation water type (surface water, rain water, ground water, drinking water)	*p* < 0.001	*p* = 0.002	*p* < 0.001
Flooding (yes/no)	*p* = 0.001	*p* = 0.010	*p* < 0.001
Average daily temperature (°C)	*p* < 0.001	*p* = 0.252	*p* < 0.001
Presence of farm animals (yes/no)	*p* = 0.444	*p* = 0.001	*p* < 0.001
Sample type (leafy greens, strawberry, water, soil, contact surfaces, seeds, fertilizer)	*p* < 0.001	*p* = 0.335	*p* < 0.001
Daily precipitation (mm)	*p* = 0.991	*p* = 0.992	*p* = 0.024
Water treatment (yes/no)	*p* = 0.200	*p* = 0.993	*p* = 0.002
Irrigation water storage type (no storage, open reservoir)	*p* = 0.051	*p* = 0.232	*p* < 0.001
Irrigation method (drip irrigation, spray irrigation, flood irrigation)	*p* < 0.001	*p* = 0.054	*p* = 0.494
Farm type (open field, greenhouse)	*p* < 0.001	*p* = 0.621	*p* = 0.053
Fertilizer type (manure-based (=raw or composted manure, pure or mixed with other types), other fertilizers (=inorganic or organic from purely vegetable origin)	*p* < 0.001	*p* = 0.418	*p* = 0.302

### 3.3. Prediction of Pathogen Occurrence Based on Significant Microbiological and Agro-Technical Factors

Multiple logistic regression was performed to investigate which factors are of major influence on the presence of pathogens when all factors are considered simultaneously, what is the extent of their impact and whether there are interactions between the significant main effects (Table 5). This analysis showed that the probability of *Salmonella* occurrence was determined by the numbers of generic *E. coli*,the country in which the data were collected, the source of the water used for irrigation water and the occurrence of a flooding event. Presence of STEC was predicted by the numbers of generic *E. coli* and the occurrence of a flooding event. Prevalence of *Campylobacter* was impacted by the country, the type of storage of irrigation water, open field farms *vs.* greenhouses and the sample type (lettuce, strawberries, water and soil).

**Table 5:** Parameter estimates of the predictors in the multiple logistic regression models for the presence (confirmed by culture isolation) of *Salmonella*, Shiga toxin producing *E. coli* (STEC) and *Campylobacter*.

Parameter	Estimation	Standard Error	95 % Confidence Interval		Significance (*p*-value)	Odds Ratio
***Salmonella* (n = 1530, *p* = 40)**						
Constant	–4.97	0.60	–6.14	–3.81	0.000	0.01
Generic *E. coli*	1.00	0.20	0.60	1.40	0.000	2.73
Spain	Reference				0.000	
Belgium	0.75	1.43	–2.05	3.55	0.600	2.12
Brazil	1.26	1.36	–1.40	3.91	0.355	3.51
Egypt	3.07	0.52	2.05	4.09	0.000	21.48
Norway	–1.54	0.82	–3.14	0.06	0.060	0.21
Surface water	Reference				0.095	
Drinking water	–15.98	2604.76	–5121.30	5089.35	0.995	0.00
Rain water	–3.27	1.37	–5.95	–0.58	0.017	0.04
Ground water	–2.61	1.09	–4.74	–0.48	0.016	0.07
Flooding	2.39	0.71	1.00	3.78	0.001	10.90
**STEC (n = 1545, *p* = 11)**						
Constant	–6.87	0.70	–8.25	–5.49	0.000	0.00
Generic *E. coli*	0.83	0.21	0.41	1.25	0.000	2.29
Flooding	1.94	0.82	0.34	3.54	0.017	6.96
***Campylobacter* (n = 476, *p* = 44)**						
Constant	–2.57	0.59	–3.73	–1.41	0.001	0.08
Norway	Reference					
Belgium	1.28	0.44	0.42	2.15	0.004	3.61
No storage of irrigation water	Reference					
Open reservoir	1.26	0.54			0.020	3.51
Farm type: open field	Reference					
Farm type: greenhouse	–1.69	0.49	–2.64	–0.74	0.001	0.18
Water	Reference				0.000	
Lettuce	–2.54	0.57	–3.66	–1.42	0.000	0.08
Strawberry	–19.89	4803.98	–9435.68	9395.91	0.997	0.00
Farm type * Water	Reference				0.042	
Farm type * Lettuce	2.18	0.86	0.48	3.87	0.012	
Farm type * Strawberry	1.69	13587.70	–26630.21	26633.59	1.000	

Note: * indicates the interaction term between two main effects.

**Figure 2:**
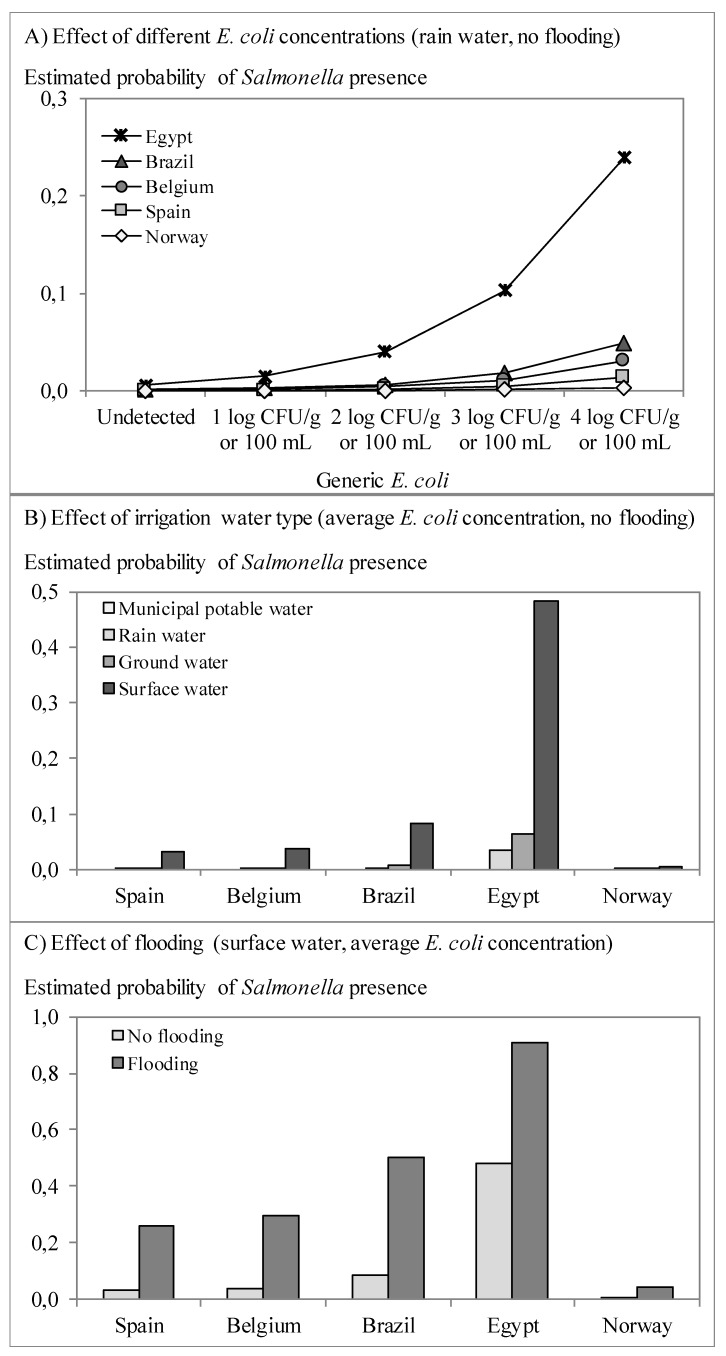
(**a**) Effect of the irrigation water type; (**b**) flooding events; (**c**) generic *E. coli* concentrations on the estimated risk of *Salmonella* presence by multiple logistic regression (Table 3).

**Figure 3:**
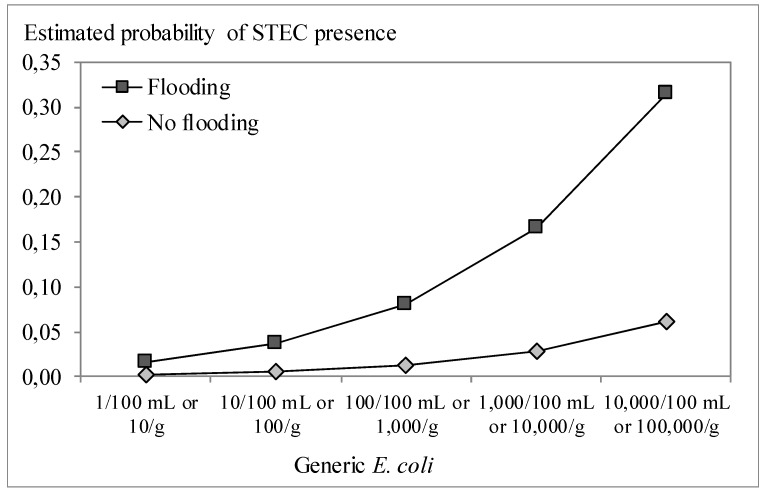
Effect of the generic *E. coli* concentration and flooding on the estimated risk of Shiga toxin producing *E. coli* (STEC) presence, isolated by culture, by multiple logistic regression (Table 3).

**Figure 4:**
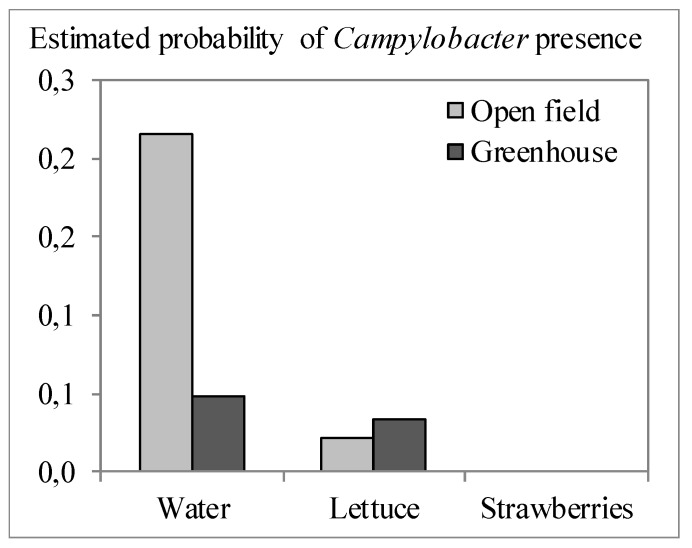
Effect of farm type and sample type on the estimated probability of *Campylobacter* presence by multiple logistic regression (Table 3), exemplified by the country Norway and the practice of not storing irrigation water in open reservoirs.

Figure 2, Figure 3 and Figure 4 graphically illustrate the results from the logistic models presented in Table 5. The prevalence of *Salmonella* and STEC was estimated to increase in case of higher generic *E. coli* counts (Figure 2a and Figure 3). The odds ratio (OR) ranged from 2.3 to 2.7, meaning that an increase of 1.0 log CFU per g or per 100 mL of generic *E. coli* doubles to triples the odds of finding pathogens. There were no interactions of *E. coli* counts with other factors, meaning that this effect applied to all countries involved in the present study and all sample types included (*i.e.*, produce, soil and water). *Salmonella* and *Campylobacter* prevalence differed significantly between countries and thus the risk estimates are specifically adjusted for each country. Detection of *Salmonella* was more likely if surface water was used for irrigation, followed by ground water, next collected rainfall water and it was least likely if municipal potable water was used (Figure 2b). Our study confirmed once more that surface water is most frequently contaminated with pathogens relative to other irrigation water sources such as rain and ground water [33,67,68]. When sampling within one week of a flooding event, the odds for *Salmonella* presence increased 10.9-fold (Figure 2c) and that for STEC 7.7-fold (Figure 3). Storage of irrigation water in open reservoirs prior to use was significantly associated with increased likelihood of *Campylobacter* detection (OR = 3.5). In particular water samples contained significantly more often *Campylobacter* than fresh produce samples (OR ≥ 12.5) and samples (of any type) taken in greenhouses showed significantly less *Campylobacter* than samples taken in open field farms (OR = 0.2), but there was an interaction between sample type and the farm type (open fields *vs.* greenhouses). This means that the ORs of sample type and farm type are not constant but vary depending on the value of the other factor. Specifically for this model, it means that the probability of finding *Campylobacter* was higher for irrigation water in open field farms than irrigation water in greenhouses, but *Campylobacter* prevalence was lower in leafy greens from open fields than leafy greens grown in greenhouses (Figure 4). Irrigation water in greenhouses presented a lower risk for *Campylobacter*, which could be explained by the more often use of reclaimed water (reuse of water after disinfection treatment) and/or the use of municipal potable water. However, the fresh produce itself grown in greenhouses seems to be more likely to finding *Campylobacter* than upon cultivation in open fields. This might be due to the exclusion of birds, lower exposure to solar UV radiation and the usually higher relative humidity in greenhouses enabling prolonged survival of microorganisms in general, and of *Campylobacter* in particular [69,70].

Risk factors for pathogen contamination could be identified but the small number of samples from which pathogens were isolated, impaired the estimation of their quantitative effects by multiple logistic regression models. Data sparseness was observed as an unequal distribution of the data over all different factor combinations. The probability of rare factor combinations was very low relative to the sample size of this study, occasionally resulting in frequencies lower than five or even zero. For example,

flooding events within one week of sampling only occurred in three out of the five individual country surveys with relatively rare frequencies (12/694 for Belgium, 36/260 for Brazil and 5/1103 for Spain), resulting in the low overall frequency of flooding of 1.8% (53/2879). Due to practical limitations in sampling and testing in the participating countries and intrinsic variability in primary production systems in place at the farms who participated on a voluntary basis in these surveys, the combined dataset was unbalanced because unequal amounts of data for all agro-technological and microbiological parameters was obtained per individual country. For example, one or two sources of irrigation water typically dominated in a specific country, with differences among the countries, resulting in partial data separation of the irrigation water sources according to country. Due to the low prevalence of pathogens in fresh produce, data sparseness issues were aggravated.

Consequently, while the (qualitative) identification of the risk factors is robust, the estimated odds ratios should be regarded as preliminary estimates, which need to be confirmed or revised after further (local or regional) data collection. Nevertheless, the identified risk factors are clearly strongly influencial risk factors on the global level which require attention in the primary production of berries and leafy greens to control and prevent the occurrence of pathogens on this fresh produce. Since the logistic regression models combined additional risk factors with the generic *E. coli* count, the predictive value for the presence of pathogens was increased in comparison to the simple use of a universal *E. coli* threshold value (larger AUC, Table 3). This shows that variable generic *E. coli* threshold values taking into account the identified risk factors can either improve the sensitivity (detecting more pathogen positive samples) or improve the specificity (reducing the number of false positives) of the performance of testing for an index organism as a surrogate for the pathogen itself. The main advantage in using the logistic regression model in comparison with solely the generic *E. coli* numbers lies in the increased specificity at a fixed sensitivity, which also translates in a higher AUC (Table 3). For example in our dataset: by setting a limit of 10 generic *E. coli* per 100 mL water, 92% of the samples containing *Salmonella* were justly rejected because they also contained ≥10 *E. coli* per 100 mL (*i.e.*, sensitivity of 92%), but at the same time 38% of the *Salmonella* negative water samples were also rejected for irrigation because they too contained ≥ 10 *E. coli* per 100 mL (*i.e.*, 62% specificity). By using additional information in the logistic regression model at 92 % sensitivity, the specificity was increased to 74% and now only 24% of the *Salmonella* negative water samples were rejected.

## 4. Conclusions

In this study, climatic parameters and factors (average daily temperature, daily precipitation and flooding of the fields) were shown to be significantly correlated with the presence of pathogens in the fresh produce production environment in univariable analysis, but with the exception of flooding, their relative importance to other microbiological (i.e. generic *E. coli* levels) and agro-technological factors (e.g., greenhouses) was too little to be retained as significant in the multivariable analysis. Other studies have identified the amount precipitation within three days prior to sampling as one of the most important risk factors for *Salmonella* detection in the fresh produce fields [71] and surface water used for irrigation [68], although the former revealed a positive and the latter a negative correlation. It should be noted that the use of weather parameters from the day of sampling may not be optimal and longer term definition of weather parameters may be more appropriate [72].

This study also showed that elevated *E. coli* numbers had moderate to good predictive value on presence of pathogens *Salmonella* and STEC, but much less for *Campylobacter*. *Campylobacter* species can reside intracellularly in protozoa such as *Acanthamoeba polyphaga,* which may allow prolonged survival and even multiplication in environmental waters. This may explain the weaker relationship with fecal indicator organisms such as *E. coli* [73]. No defined number of generic *E. coli* in for example strawberries, leafy greens or water was shown to serve as a threshold value to distinguish between safe and unsafe produce or irrigation water. Instead it was shown that taking into account the status of defined risk factors (*i.e.*, the country of sampling, the sample type, a flooding event) will enhance the functionality of predicting the presence of pathogens in fresh produce and could contribute to more efficient and risk-based testing for index organisms (or pathogens) in the quest to ensure safety of the fresh produce. It is however recommended that further data are collected in the various regions of the world with regard to microbiological quality of fresh produce and the production environment to further underpin and confirm the results of the present study in relation to risk factors and their estimated (quantitative) impact on safety of the fresh produce. It is known there is considerable variation in weather conditions over the years which may influence the microorganisms in the agricultural environment [74]. In addition, geographic regions differ in their organization and management of the fresh supply chain which will also impact on the finding of risk factors. Moreover, to which extent the risk factors have been tackled already by defined control procedures and assurance activities (including microbiological monitoring) in place varies considerably on a global level. The relation of *E. coli* with pathogens is complex, whether *E. coli* may function as a suitable index organism or not depends on the pathogen, the climate and seasonality, the geographic region, the sample type (soil, water, fresh produce) and the presence of animal and human reservoirs, which is illustrated by the fact that contradictory results have been obtained in previous studies [31,68,75,76,77,78].

In conclusion, this study combined data sets from different countries but equivalent sampling plans and contributed to the better understanding of key factors on a global level that need attention in good agricultural practices on the farm. This study also showed testing for *E. coli* numbers can provide information on the likelihood of finding pathogens and thus serve as an index organism to reliably assess food safety of fresh produce, testing and sampling needs to be driven by information on adoption of food safety practices, local weather conditions and incidents, which may vary upon the regional location of the farm.
